# Protective Intranasal Immunization Against Influenza Virus in Infant Mice Is Dependent on IL-6 

**DOI:** 10.3389/fimmu.2020.568978

**Published:** 2020-10-28

**Authors:** Elizabeth Ann Bonney, Kendall Krebs, Jihye Kim, Kirtika Prakash, Blake L. Torrance, Laura Haynes, Mercedes Rincon

**Affiliations:** ^1^Department of Obstetrics, Gynecology and Reproductive Sciences, Larner College of Medicine, University of Vermont, Burlington, VT, United States; ^2^Division of Medical Oncology, Department of Medicine, University of Colorado, Anschutz Medical Campus, Aurora, CO, United States; ^3^Department of Immunology, University of Connecticut Center on Aging, Farmington, CT, United States; ^4^Division of Immunobiology, Department of Medicine, Larner College of Medicine, University of Vermont, Burlington, VT, United States; ^5^Department of Immunology and Microbiology, University of Colorado Anschutz Medical Campus, Aurora, CO, United States

**Keywords:** infant, influenza, mouse, vaccination, lung

## Abstract

Respiratory diseases adversely affect infants and are the focus of efforts to develop vaccinations and other modalities to prevent disease. The infant immune system differs from that of older children and adults in many ways that are as yet ill understood. We have used a C57BL/6 mouse model of infection with a laboratory- adapted strain of influenza (PR8) to delineate the importance of the cytokine IL-6 in the innate response to primary infection and in the development of protective immunity in adult mice. Herein, we used this same model in infant (14 days of age) mice to determine the effect of IL-6 deficiency. Infant wild type mice are more susceptible than older mice to infection, similar to the findings in humans. IL-6 is expressed in the lung in the early response to PR8 infection. While intramuscular immunization does not protect against lethal challenge, intranasal administration of heat inactivated virus is protective and correlates with expression of IL-6 in the lung, activation of lung CD8 cells, and development of an influenza-specific antibody response. In IL-6 deficient mice, this response is abrogated, and deficient mice are not protected against lethal challenge. These studies support the importance of the role of the tissue environment in infant immunity, and further suggest that IL-6 may be helpful in the generation of protective immune responses in infants.

## Introduction

Respiratory tract inflammatory and infectious disease in infant children represents a significant burden to the healthcare system (1, 2). A particular example is influenza virus infection, for which there is evidence of increased incidence of disease severity in infants, as compared to adults in humans and animal models (3, 4). Classic theory suggests that adaptive immunity, predominantly the T cell immune response, is altered in infants in order to support self-tolerance, maternal tolerance, and tolerance to new developmental or environmental antigen (5–7). More recent thinking about infant immunity considers the possibility of distinct populations of T cells present in the infant, but not in the adult, that display a “tolerance” phenotype (8). Alternative models suggest that T cells in neonates/infants are not inherently tolerant, but that tissue specific signals can affect T cell activation and effector function by limiting or supporting the access to productive antigenic signals (9–17).

The role of the tissue environment (independently of the immune cells) in which specific immune responses occur could also be an important factor that affects the immune response in neonates/infants (10, 18). Specifically, for influenza virus and other respiratory infections, the epithelium of the respiratory tract provides a unique environment that can tune adaptive immune responses. Lung epithelial cells are a major source of type I IFN which plays a critical role in antiviral responses (19). In addition, lung epithelial cells can produce a variety of inflammatory cytokines (e.g., IL-6) that can influence the course of T cell responses (19). Lung epithelial cells in infants differ from those in adults in humans (20–23) and animals (24). The potential effect of lung epithelial cells in the immune responses of infants is a recent area of inquiry, and could be a significant determining factor in their apparent altered immune response during influenza virus infection.

Although intramuscular administration of a polyvalent formulation of chemically inactivated or disrupted influenza virus is the most common type of vaccine for seasonal influenza both in adults and children (25), intranasal administration of a live attenuated influenza virus (LAIV) in the respiratory tract is also utilized as a method of vaccination (26). However, this formulation is restricted to children more than 2 years of age and adults (27, 28) because of reports of increased risk of reactive airway disease (29, 30) and increased concern over the presence of underlying asthma in young vaccine recipients (31, 32). The factors underlying the relatively lower effectiveness of recent intranasal vaccination with live attenuated virus compared to intramuscular administration of inactivated virus in some flu seasons (33–35) remain incompletely understood. Together, these issues elevate the question of whether there might be novel specific mechanisms or alternative approaches to enhance influenza vaccination in the very young. Similar to the response to respiratory virus infection, lung epithelium could also play a key role in determining the type and strength of the immune response that intranasal immunizations can trigger.

Interleukin 6 (IL-6) is a member of a family of cytokines that play an important role in both innate and adaptive immune responses (36). In addition, IL-6 is important in the processes of tissue regeneration and inhibition of apoptosis (37). IL-6 is produced by innate immune cells such macrophages, but it is also produced by a variety of cell types (e.g. epithelial cells, endothelial cells, astrocytes) upon exposure to insults (36). For example, we and others have shown that IL-6 is produced by lung epithelial cells in response to viral infection or allergens (38, 39). In adult mice, IL-6 can enhance T-cell mediated antibody response against i.m. influenza immunization (40) and there is evidence supporting the importance of IL-6 in the early response to influenza infection (41). However, the specific importance of IL-6 in infant immunity to influenza is less clear (42).

Here, we have performed gene expression profiling studies comparing lung epithelial cells from infant and adult mice and the results have revealed a compromised expression of IL-6 and related signaling pathways in infant lung epithelial cells. Using a heat-inactivated influenza virus, as a surrogate for current attenuated virus formulations, we show that i.m. administration in infant mice does not provide protection, while intranasal administration does. However, such protection induced by i.n. immunization is dependent on IL-6. Thus, these studies underline the relevance of the tissue environment for the efficacy of vaccinations in infants, and they bring to light potential mechanisms related to differences in infant and adult lung epithelium that could influence the efficacy of immunizations. Our results could be relevant for future improvement of vaccines for young children.

## Material and Methods

### Mice

C57BL/6J (wild type, WT obtained from Jackson Laboratory) or IL-6 KO mice (43) were housed under specific pathogen free, AAALAC-approved conditions using a 12-h light cycle and were given food (normal Chow) and water ad libitum. IL-6 KO mice used in these studies were backcrossed over 12 generation with C57Bl/6J. Females of both strains and 8-24 weeks of age mated freely with same strain males and littered without interference. Thirteen to fourteen days after birth, while still nursing, mothers and infant mice moved to a biosafety room, where they acclimated for 12–24 h before infection or immunization of the pups.

### Influenza Virus Infections and Immunizations

These studies utilized Puerto Rico A/PR/8/34 H1N1 influenza A (PR8) (41). Intranasal and intramuscular immunizations utilized heat inactivated PR8 influenza virus (iPR8). Inactivation was performed by incubation at 56 degrees for 30 min. This method of inactivation allows production of a virus that enters cells, since it does not fully denature the HA protein of the virus, but which does not replicate since the virus polymerase is made inactive at this and lower temperatures (44, 45). iPR8 (5 × 10^5^ EIU) in 15–20 or 50 µl PBS was used for intranasal or intramuscular immunizations, respectively. The average weight was 5.9+/− 0.15 g at 14–16 days of life and this was not statistically different from any experimental group (**Supplementary Figure S1**). In two experimental cohorts, mice were euthanized three weeks after immunization to collect tissues for *in vitro* examination of the immune response to immunization.

For lethal challenge in juvenile mice (day 35–48 of life) we used 6 × 10^3^ EIU PR8 given intranasally in 50 µl of PBS. For the batch of PR8 virus used in these studies, 10^4^ EIU corresponded to 2 LD50 when initially tested in adult mice (8–10 weeks of age). For juvenile mice between 30–45 days of age, we found 6 x 10^3^ EIU PR8, could be equivalent to the 2 LD50 in adult mice (**Supplementary Table I**). Female mice weighed 16±1gram and males weighed 20±1gram at the time of challenge. Mice underwent inhalant anesthesia (1.5 L/min O2, 2% isoflurane) in a chamber connected to an isoflurane vaporizer to receive intranasal immunizations or infections with live virus. Infected mice were weighed every 24–48 h. Mice reaching below 70% of starting weight were euthanized consistent with stipulations of our animal use protocol (University of Vermont IACUC# 13-029). Therefore “survival” in these studies indicates mice who did not fall below this threshold. Though males of each group at challenge were heavier than same strain females, the weights of WT and IL6KO females and those of WT and IL6KO males were comparable (**Supplementary Figure S2**). To account for male and female weight differences, males were challenged with higher doses. **Supplementary Figure S3** shows a representative sample of the weight of WT females who were challenged with virus.

### Determination of Influenza Viral Load in Tissues

Lungs from assayed mice were freshly harvested and frozen in liquid nitrogen. RNA was isolated from whole lung tissue homogenized in TRIzol reagent (Invitrogen Life Technologies). cDNA was synthesized using iScript cDNA synthesis kit (Bio-Rad Laboratories), using the manufacturer’s protocol. Viral loads in harvested whole lungs were determined by real-time RT-PCR for the PR8 viral acid polymerase (PA) gene by comparison to a standard titration of viral PA copies run on the same PCR assay, with 20ng of cDNA used per reaction. The following primers and probe were used to amplify and quantitate the PR8 PA gene: forward primer, 5′-CGGTCCAAATTCCTGCTGA-3′; reverse primer, 5′- CATTGGGTTCCTTCCATCCA-3′; probe, 5′-6-FAM-CCAAGTCATGAAGGAGAGGGAATACCGCT-3′ (Integrated DNA Technologies) (41).

### Cytokine Gene Expression

In alternate RNA isolation protocols, the Qiagen RNeasy Mini kit (PN 74104) was utilized as recommended by the manufacturer. cDNA was synthesized as above. Relative mRNA levels were determined by qRT-PCR using Assays–on–Demand TaqMan Gene Expression Assays (FAM-MGB, ThermoFisher Scientific https://www.thermofisher.com) for IL-6(Mm00446190), CCL2(Mm00441242), γIFN(Mm01168134), IL-10(Mm01288386), TNF(Mm00443258), TGFB1 (Mm01337605), and Beta-2 microglobulin (Mm00437762). Values reported are those obtained after normalization to β2–microglobulin and analyzed by the comparative delta CT method. In addition, serum cytokines were quantified using a Luminex ® xMAP® multiplex platform, combined with a customized Milliplex™ mouse chemokine/cytokine panel from Millipore™.

### Analysis of Anti-Influenza Virus Specific Antibodies in Serum by ELISA

Influenza-specific antibody levels in serum samples were determined by ELISA, as previously described (40). ELISA plates were coated with inactive influenza PR8 virus (10^7^ EIU/ml) in sodium bicarbonate buffer, washed, blocked (1% BSA/PBS solution) and incubated with 2-fold serial dilutions of serum overnight. Plates were washed and incubated with HRP-conjugated goat anti-mouse total IgG (SouthernBiotech) for 45 min at room temperature. Plates were then washed and developed using TMB Sureblue substrate and development was stopped with TMB stop solution (ThermoFisher). Plates were read at 450 nm in a plate reader.

### Flow Cytometry Analyses of Lungs

Whole lungs harvested from immunized mice were used to prepare single cell suspensions using the gentle MACS™ (Miltenyi Biotech) tissue dissociation system. Red cells were removed with Geyes lysis medium and the resulting cell suspensions were washed in Iscove’s Modified Dulbecco’s medium with 5% FBS. Cell suspensions were stained with antibodies to CD45 (CD45.2, clone 104, PerCP-Cy™ 5.5), CD8(CD8α clone 53–6.7, Pacific Blue™) CD4 (clone GK1.5, R-phycoerythrin) and CD44 (clone IM7 fluorescein isothiocyanate) and were run on an LSRII (BD Biosciences). The gating scheme for these studies is shown in **Supplementary Figure S5**.

### Lung Epithelial Cell Gene Expression Analysis

Four samples, each consisting of pooled epithelial cells from the lungs of three male pups or three female pups aged 14 days (total eight pups samples), or epithelial cells from four individual male and four female adult (eight weeks) lungs (eight adult samples total) were used to isolate RNA and examined by array transcriptome profiling. Mice were euthanized by cervical dislocation. Lungs were removed under sterile conditions into 1X PBS and cut up into very small pieces. Tissues were transferred to MACS C-tubes (purple tubes) for homogenization using the gentle MACS™ (Miltenyi Biotech) tissue dissociation system. Red cells were removed by treatment with Geye’s solution, and this was followed by resuspension in DMEM/F12+ 5% FBS. The resulting cell suspension was incubated in a cell culture plate at 37C degrees and 5% CO2. Afterwards, nonadherent epithelial cells were removed by slowly rocking the plate back and forth and gently removing the supernatant. This was centrifuged, resuspended and washed in MACS™ buffer and then incubated with anti-CD45 (Miltenyi) beads to remove CD45 + cells by passing the incubated solution on a magnetized LS column (Miltenyi). Purity was checked by flow cytometry for CD45 (less than 5 %) and histochemical identification of keratin + cells. Transcriptome profiling was done using the Affymextrix GeneChip system (Mouse Gene 2.0 ST Array). Chip quality was verified, and scan data was analyzed using RMA (46). Analysis of array data was performed using Partek Genomics Suite® 6.6. Beta Analysis. We report comparisons between groups using the number of probe sets that pass an FDR of 0.05, or a binary filter (p<0.05 and 2x fold change).

### Functional Analysis

We used GSEA (Gene Set Enrichment Analysis) and pathway analysis, an approach that offers an unbiased global search for genes that are coordinately regulated in predefined pathways (47) rather than interrogating expression differences of single genes. Gene set analysis was performed using the GSEA software (48) version 4.0.3 obtained from https://www.gsea-msigdb.org/gsea/downloads.jsp. The gene sets database was compiled from the Kyoto Encyclopedia of Genes and Genomes (KEGG) database http://www.kegg.jp/ (47). The KEGG gene sets database contains 210 mouse pathways that include metabolism, genetic information processing, environmental information processing, cellular processes, and human diseases. One hundred eight-eight gene sets passed the gene set size filter criteria (min, 10; max, 500). *P* values for the gene sets were computed by permuting the gene sets 1,000 times in this study.

### Additional Statistical Analysis

Viral load, cytokine levels, and antibody level were compared using one-way ANOVA or nonparametric analysis as appropriate. Due to small numbers, most normality testing used the Shapiro-Wilk test. Survival analysis with threshold being weight below 70% of starting (point at which we were compelled to euthanize mice) was performed using the log-rank (Mantel-Cox) test. Data shown represents survival analysis of combined data of percentage of initial weight over time from challenge from over 8 cohorts of mice that received immunization and or challenge as indicated. Means± SEM or Median with range are reported, depending on the hypothesis test used. For hypothesis testing, significance was set at p<0.05.

## Results

### High Mortality to Sublethal Dose of Influenza Virus in Infant Mice

**Figure 1:**
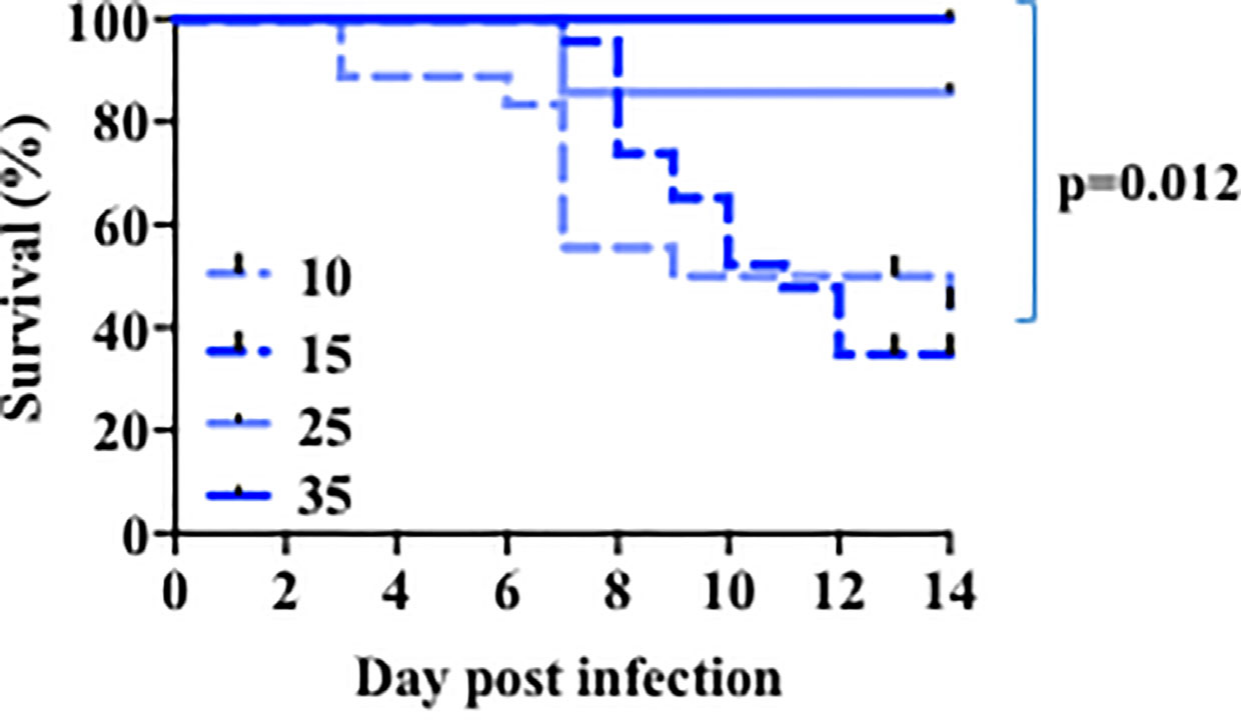
Age-related susceptibility to a sublethal dose of PR8 influenza virus. The 10 days-old mice group (n=18) received 140-200 EIU (~30 EIU per gram of weight) of live PR8 influenza virus, the 15 days-old mice group (n=23) received 200 EIU (~30 EIU per gram of weight), the 25 days-old mice group (n=7) received 300–900 EIU (~30 EIU per gram of weight), the 35 days-old mice group (n=6) received 3000 EIU (~ 200 EIU per gram of weight). Survival was followed. Solid lines, older mice. Dotted lines, infant mice. p value, comparison of curves for day 15 and 35 by Mantel-Cox test. Analysis is of total data obtained from 12 independent cohorts of mice.

Similar to humans, infection with a sublethal dose of influenza virus in adult mice leads through a period of sickness during the peak of virus replication, and eventual recovery upon virus clearance from the lung. Several lines of evidence suggest that children less than five years of age are more susceptible to seasonal influenza virus infection (3, 49) and this led us to first investigate the age-related susceptibility of infant mice to influenza. We performed viral infection in mice 10–44 days old with different doses of PR8 influenza virus. Because the size of the lungs is determined by the body size of the mouse, we used different doses to normalize by weight. As we have shown (41), nearly all young adult (~5 weeks of age) mice survived infection with a sublethal dose of influenza (**Figure 1** and **Supplementary Table 1**). When we infected 24-day-old mice with same viral dose/weight ratio, we observed a small fraction of mortality (**Figure 1** and **Supplementary Table 1**). In contrast, infection of 10 day or 14–16-day-old mice with a comparative viral dose/weight ratio caused ~75% mortality (**Figure 1** and **Supplementary Table 1**). Thus, when corrected for body size, infants are highly susceptible to an otherwise sublethal dose of PR8 virus infection.

### Intranasal, but Not Intramuscular, Immunization With Inactive Influenza Virus Provides Protection in Infant Mice

**Figure 2:**
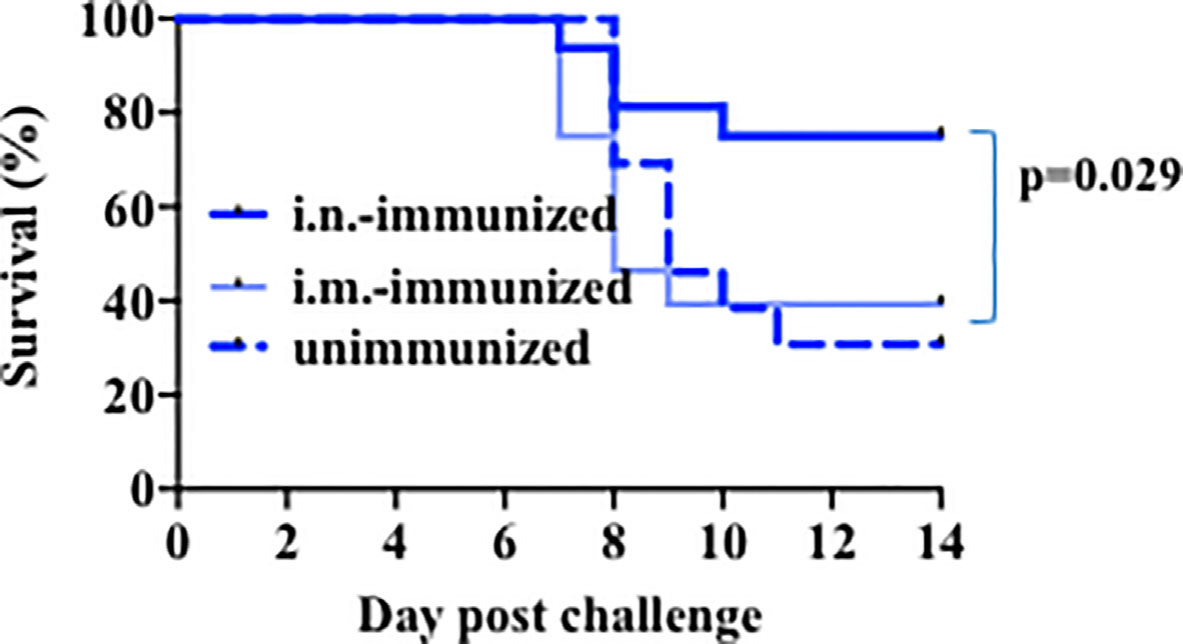
Intranasal immunization with heat inactivated virus protects infant mice against lethal challenge. Mice aged 14–17 days of life were immunized intranasally (Bold Dk blue, n=16) or intramuscularly (Lt blue, n=28) with 5 × 10^5^ EIU heat inactivated PR8 virus, or left unimmunized (dotted lines, n=13). Three weeks later, all mice received a lethal dose of live virus. Y axis: Survival. Mantel-Cox (log-rank) analysis was used to compare unimmunized to i.n.-immunized mice. Significance was set at p<0.05. Analysis is of total data obtained from 8 independent cohorts of mice receiving immunization and or challenge.

Because very young children, like infant mice examined above, are more susceptible to death with influenza virus, there has been pressure to identify the most appropriate and efficacious way to immunize them against influenza. However, existing practical, clinical, and biologic limitations on the type of influenza vaccine that is currently provided to very young children make this issue a subject of intense investigation. We therefore used our mouse model above to determine a route and format of immunization that could protect infant mice against lethal infection. Because most children only receive an intramuscular injection of influenza vaccine, often with minor benefit (50), we compared both intramuscular (i.m.) and intra-nasal (i.n.) modes of vaccination. Two-week-old mice were immunized with heat-inactivated PR8 virus (5 × 10^5^ EIU/mouse) through i.m. or i.n. administration. As a control, we also included a group of infant mice that received no immunization. Three weeks post-immunization, mice were challenged with a lethal dose of influenza PR8 virus. The mice were observed for weight loss and clinical signs of severe illness as parameters to determine mortality. Mortality in mice immunized by i.m. administration was comparable to the mortality of mice that received no immunization (**Figure 2**), indicating that i.m. immunization does not provide protection from a lethal dose of PR8 virus. In contrast, relative to i.m. immunized and unimmunized mice, those mice immunized through i.n. administration of the same inactive PR8 influenza virus have better survival to the infection with the lethal dose of influenza virus (**Figure 2**). Thus, i.n. administration of the inactive virus provides superior protection in infant mice to influenza virus infection.

### Gene Expression Profile Analysis Reveals the Attenuation of IL-6 Gene Expression in Lung Epithelial Cells for Infant Relative to Adult Mice

The increased protection obtained through an intranasal administration of iPR8 relative to the lack of protection with the intramuscular administration suggests that there is some specific component in the lung which supports the immune response. In addition, the fact that infants are more susceptible than adult mice to primary influenza infection (**Figure 1**), suggested that there may be tissue specific differences, the adaptive immune response notwithstanding, that are critical for resolution of infection in infant as compared to older mice. Lung epithelial cells are capable of producing a number of cytokines in response to different insults (e.g. viral infection) (19). To investigate the presence of potential underlining differences between infant and adult lung epithelial cells we performed microarray analyses. We used RNA from lung epithelial cells freshly isolated from naïve infant (2 weeks-old mice) and adult (8 weeks-old mice) mouse lungs. Each pup sample (eight total, four male and four female) included pooled lung epithelial cell RNA preparation from 3 pups, while each adult sample (eight total, four male and four female) contained RNA from a single adult.

Analyses of the microarray results revealed that the number of probe sets that pass an FDR of 0.05 as being differentially expressed in infant versus adult epithelial cells was 7,334. Those passing a binary filter (FDR <0.05 and 2x fold change) were 724. Gene Set Enrichment Analysis and the KEGG database were used to determine pathways that were significantly differently expressed in infant versus adult cells using a criterion of p-value (<0.05) and NES (> |1.5|).

**Figure 3:**
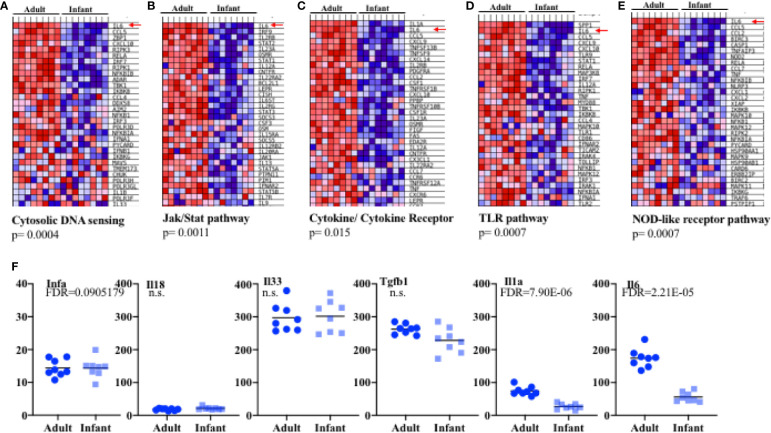
Expression profiling in epithelial cells from infant and adult mice reveals IL-6 deficiency at baseline. Four samples each consisting of pooled epithelial cells from the lungs of three male pups or three female pups aged 14 days (total eight pups samples) or epithelial cells from four individual male and four female adult (8 weeks) lungs (eight adult samples total) were used to isolate RNA and examined by array transcriptome profiling. Gene Set Enrichment Analysis (eight total pools per group) revealed significant differences in expression in pathways relevant to innate immunity. **(A–E)** Shown are heat maps for the most highly differentially expressed genes within in the various pathways. Red-up regulated, blue down regulated. Red Arrows: IL-6. *P* values for the gene sets were computed by permuting the gene sets 1,000 times. **(F)** Transformed RMA data for specific genes in infant versus adult epithelial cell pools. Each symbol refers to a pool of 3 mice. FDR, fold discovery rate (reflecting comparison of groups of pools with modification for multiple comparison testing as part of the array analysis) was calculated using Partek Suites Genomics® 6.6 Beta Analysis.

We focused further attention on those immune-related pathways that might be relevant to influenza and that were significantly of lower expression in infant cells as compared to adult. Several pathways were identified. One of the pathways markedly (p= 0.0004) lower in infants was the “cytosolic DNA-sensing pathway” that includes host genes involved in sensing bacteria and viruses such as members of the inflammasome pathway, RIG pathway, type I IFN, chemokines, NF-kB and some cytokines (**Figure 3A** and **Supplementary Figure S6A**). Within-pathway analysis defined a cluster of genes with lower expression in epithelial cells from the lungs of infants relative to adult mice (**Figure 3A**, **Supplementary Figure S6A**). Interestingly, IL-6 was the gene most significantly lower in lung epithelial cells from infants (**Figure 3A**, **Supplementary Figure S6A**). Upon binding to its receptor, IL-6 activates the Jak/Stat pathway leading to the activation of Stat3 (51). The KEGG “Jak/Stat pathway” was also substantially under-expressed in lung epithelial cells from infants relative to adult mice (**Figure 3B**, **Supplementary Figure S6B**). This pathway includes genes for cytokines, cytokine receptors, transcription factors, kinases etc. Among them, IL-6 was on the top of the cluster of genes that were significantly lower in epithelial cells from infants (**Figure 3B**, **Supplementary Figure S6B**). The “Cytokine-cytokine receptor interaction” pathway, containing a number of genes for cytokines/chemokines and their receptors, was also observed to be significantly decreased and IL-6 together with IL-1 were the genes with the lowest expression in infant lung epithelial cells relative to the expression found in adult mice (**Figure 3C**, **Supplementary Figure S6C**). Two other innate immune pathways were identified to be significantly lower in infants. One was the “TLR signaling pathway” that includes pattern recognition receptors responsible for detecting microbial pathogens and generating innate immune responses including molecules such as type I IFN, NF-kB, and inflammatory cytokines (**Figure 3D**, **Supplementary Figure S6D**). Another was the NOD-like receptor (NLR) signaling pathway that includes intracellular NLR family members, cytokines regulated by this pathway, caspases, NF-kB, and others (**Figure 3E**, **Supplementary Figure S6E**). Of interest, among all the different genes included in these two pathways, IL-6 was identified as the gene most reduced in lung epithelial cells in infants (**Figures 3D, E**, **Supplementary Figures S6D, E**). Additional comparative modeling for specific cytokines known to be produced by lung epithelial cells further demonstrated selectively reduced expression of IL-6 in infant cells (**Figure 3F**). We did not observe significant differences in the expression of innate cytokine genes well-known to be expressed in epithelial cells (e.g., IFNa, IFNb, IL-33, IL-18, **Figures 3C, F**, **Supplementary Figure S6C**). We have previously shown a constitutive expression of IL-6 (high levels of IL-6 mRNA) in lung epithelial cells isolated from adult wildtype mice under physiological conditions prior to any exposure or insult (38), while no expression was detected in resident leukocytes (CD45^+^ cells) (38). The results here show that IL-6 gene expression in lung epithelial cells is regulated during development and its expression is attenuated in infants.

### Protection of Infant Mice by Intranasal Immunization With Inactive Influenza Virus Requires IL-6

**Figure 4:**
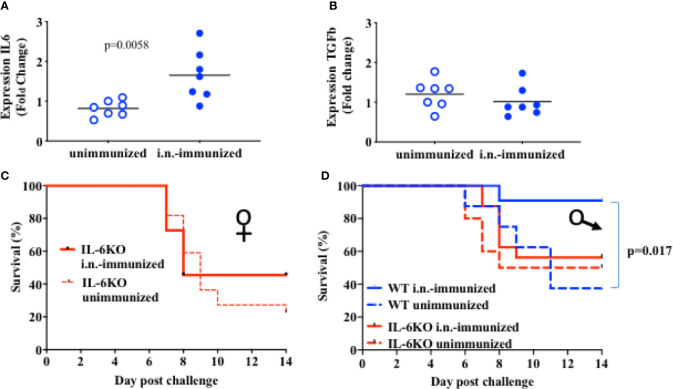
IL-6 is necessary for protective response to an i.n. administration of heat inactivated influenza virus. **(A, B)**: On day 16 of life mice received 5 × 10^5^ EIU of heat-inactivated virus intranasally (Dk blue) or not (Lt blue). At 2 days after immunization, lungs were harvested and whole lung was used to extract RNA that was later assayed for RNA expression of IL-6 **(A)** and for TGFb **(B)** by QPCR. Each symbol represents one mouse. Shown is fold-change relative to unimmunized mice in a single experiment. The t-test was used to test for significant differences between groups. **(C, D)**: Mice aged 14–17 days of life were i.n.-immunized (bold) with 5 × 10^5^ EIU heat inactivated virus or left unimmunized (dashed). Three weeks later, all mice received a lethal dose of live virus. Y Axis, survival. X axis, day post challenge. **(C)** IL-6KO Female mice; i.n.-immunized, n = 11; unimmunized, n = 22. **(D)** Male mice; IL-6KO (red) i.n.-immunized, n = 16; unimmunized, n = 10. WT males (blue) i.n. immunized n = 11, unimmunized, n = 8). Shown is Mantel-Cox (log-rank) analysis of total data using % initial weight as noted in methods from 5 independent cohorts of mice receiving immunization and or challenge. Significance was set at p < 0.05.

Administration of IL-6 has been shown to enhance the effectiveness of a subcutaneous inactive influenza virus vaccine in adult mice (40). IL-6 derived from lung epithelial cells could therefore contribute to the protective effect of intranasal vaccines. Since the basal levels of IL-6 gene expression in lung epithelial cells in infants was significantly lower when compared to adults, we examined whether i.n. administration of the inactive influenza virus vaccine could upregulate IL-6 expression. Two-week-old mice were administered with an i.n. dose of inactive PR8 virus and the lung was harvested 2 days post-immunization for cytokine expression. Relative to the levels in lung from non-immunized mice, higher levels of IL-6 expression were present in lungs from i.n.-immunized mice (**Figure 4A**). In contrast, we did not observe an increase in other cytokines, such as TGFβ, in lungs from immunized infants (**Figure 4B**). Thus, i.n. administration of an inactive influenza virus selectively induced IL-6 expression, suggesting that local production of IL-6 in the lung could contribute to the protective effect of i.n. administration relative to i.m. administration.

We next investigated the contribution of IL-6 to the protective effect of i.n. immunization with inactive influenza. IL-6-deficient infants (2 weeks-old) were i.n.-immunized with inactive PR8 virus as described above for wildtype mice, or mice were left with no immunization. Three weeks post-immunization, both immunized and non-immunized mice were challenged with a lethal dose of PR8 virus. Intranasal immunization in IL-6-deficient mice resulted in mortality similar to that observed in unimmunized mice (**Figures 4C, D**) with more than 50% of mice dying by 10 days after challenge. This was true for both females (**Figure 4C**) and males (**Figure 4D**). In contrast to WT female mice (**Figure 2**) and WT male mice (**Figure 4D**), i.n. administration of inactive PR8 fails to provide protection in IL-6 KO mice, suggesting that the success of an i.n. immunization with inactive virus relies significantly on IL-6 production in the lung.

### Lack of IL-6 Does Not Cause an Exuberant Systemic Immune Response to Influenza Virus Infection

**Figure 5:**
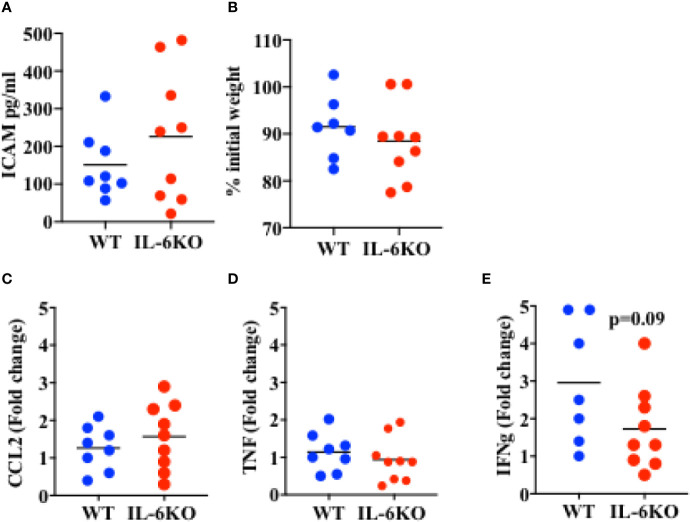
Response to a lethal dose of influenza in i.n. immunized WT and IL-6KO mice. Mice received intranasal immunization with inactive virus on day 14 of life. Three weeks later, mice were weighed and given a lethal dose PR8 virus. Six days after challenge mice were euthanized and tissues harvested for analysis. **(A)** Serum ICAM was analyzed by Luminex™. **(B)** Weight of mice, 6 days post-infection. Shown is the % relative to the initial weight prior to infection. **(C–E)** Whole lung relative mRNA expression for CCL2 **(C)**, TNF **(D)** and IFNg **(E)** determined by real time RT-PCR. Values show fold induction relative to a WT. Each dot represents a mouse. One experiment is shown. Bars indicate mean for group. Comparison utilized the t test, with significance set at p < 0.05.

The results above show that i.n.-immunized IL-6 KO infants experience significant mortality in response to a lethal dose of PR8 influenza virus, while immunized WT infants are significantly protected. The ultimate increased mortality in IL-6-defficient mice could be due to an early-post-challenge exuberant and dysregulated innate response, leading to systemic tissue damage and death. Conversely, our findings could be explained by an insufficiency in the adaptive and protective immune response generated by immunization. To attempt to differentiate between these two possibilities, we investigated the systemic and local immune responses after viral challenge. WT and IL-6 KO mice were intranasally immunized with an inactive PR8 virus as described above. Three weeks post-immunization, mice were infected with a lethal dose of PR8 virus and lungs and serum were harvested 6 days post-infection. Lung viral loads in WT and IL6KO mice were similar (**Supplementary Figure S4**), suggesting that there were not significant differences in the initial ability to undergo infectious challenge. The levels of soluble ICAM in serum, a marker of systemic inflammatory response, endothelial cell activation and acute respiratory distress (52, 53), were comparable between WT and IL-6 KO mice (**Figure 5A**). In addition, there was no difference in weight loss between WT and IL-6 KO mice at this early time point of the infection (**Figure 5B**). Since it has been reported that IL-6 deficiency could affect macrophage recruitment by affecting chemokine expression (54), we examined CCL2, important for monocyte trafficking, but no difference was found between WT and IL-6 KO mice (**Figure 5C**). Further, analysis of cytokine expression in the lungs revealed no difference in inflammatory markers such TNF (**Figure 5D**). However, the analysis of IFNγ, a product of the adaptive immune response (CD4 and CD8 T cells), showed a trend toward reduced expression in immunized IL-6 KO mice after the lethal infection with influenza virus (**Figure 5E**). Thus, these results suggest that the death of immunized IL-6 KO infants in response to influenza virus is not caused by an enhanced early pathogenic and dysregulated innate immune response, but they instead suggest an impaired memory T cell response.

### IL-6 Is Required for Intranasal Immunization With Inactive Influenza Virus to Sustain Memory T Cell Response in Infant Mice

**Figure 6:**
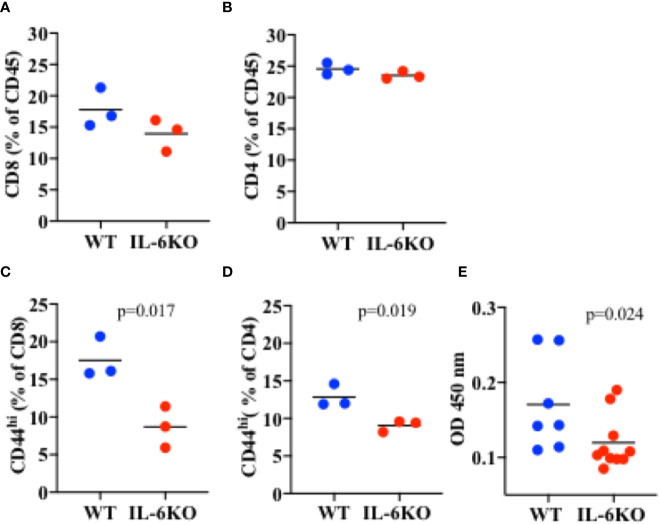
Lower frequency of memory CD8 and CD4 cells in the lungs of i.n. immunized IL-6 KO mice. Mice (n = 3) received intranasal immunization with inactive virus on day 14 of life. Three weeks later, lungs were extracted and analyzed by flow cytometry. **(A)** Cells were gated for CD45 first. % of CD8 cells within the CD45+ population is showed. **(B)** Cells were gated for CD45 first. % of CD4 cells within the CD45+ population is showed. **(C)** Cells were gated for CD45 first, and then for CD8 cells. % of CD44^high^ within CD8 cells is shown. **(D)** Cells were gated for CD45 first, and then for CD4 cells. % of CD44^high^ within CD4 cells is shown. p value is determined by t test. **(E)** PR8-specific IgG antibody titer in serum determined by ELISA. p value is calculated using the Mann-Whitney test, with significance set at p < 0.05. One experiment is shown.

To investigate whether, in infants, IL-6 is required for an intranasal vaccine to trigger an efficient adaptive memory immune response, 2-week-old WT and IL-6 KO mice were immunized intranasally with inactive influenza virus as described above. Three weeks post-immunization, lungs from immunized mice were harvested and processed, and different T cell populations in lung cell homogenate were examined by flow cytometry analysis. Leukocytes were first gated from other cell populations in the lung using CD45 as a pan leukocyte marker (**Supplementary Figure S5**). We examined the presence of CD8 and CD4 cells within the CD45 cell population. No significant difference in the percentage of CD8 cells could be detected between WT and IL-6 KO i.n.-immunized mice (**Figure 6A**). Similarly, no significant difference in the presence of CD4 cells was found between WT and IL-6 KO mice (**Figure 6B**). However, when we examined the presence of memory CD8 cells using CD44^high^ as marker, the frequency of memory cells was markedly reduced in lungs from immunized IL-6 KO infants relative to WT infants (**Figure 6C**). Similar results were obtained for CD4 cells. The frequency of CD4 CD44^high^ cells was significantly lower in IL-6 KO infants relative to WT mice (**Figure 6D**). Thus, during intranasal influenza immunization of infants, IL-6 does not seem to promote the recruitment of lymphocytes, but is important in a sustained memory T cell response.

IL-6 has been shown to promote antibody production indirectly by acting on CD8 and CD4 T cells and supporting their IL-21 production. IL-21 in turn acts on B cells to promote antibody production, primarily IgG (40, 55). We therefore also examined the presence of influenza-specific IgG in the serum of WT and IL-6 KO infants 3 weeks post-immunization. The levels of PR8-specific IgG were reduced in immunized IL-6 KO infants compared with WT mice (**Figure 6E**). Thus, during i.n. immunization of infants, IL-6 plays an important role in sustaining effective T and B cell adaptive memory.

## Discussion

Infant immunity remains an important area of focus due to significant existing morbidity related to infectious disease. Influenza infection remains a major challenge among infections in children under five years of age (56). While historically it is believed to be due to an inappropriate T/B cell immune response, pathology and resolution of primary influenza virus infection is not dependent on the adaptive immunity but requires innate immunity (57). Another potential difference between children and adults is dose of the virus to which they are environmentally exposed relative to lung size (again, determined by body size). However, in our studies here we show that even when influenza viral dose is normalized to body weight (reflecting lung size), infant mice are more susceptible to sublethal dose than adult mice. Therefore, an increased virus dose/lung size ratio does not seem to be the main cause for the enhanced sensitivity to influenza virus response in infants. This disparity seems to be a developmental issue. Here, our Microarray studies have revealed a different gene expression profile in epithelial cells from the lungs of adult and infant mice prior to exposure to any infection or other type of insult.

The view that infant immune cells are inherently deficient contrasts with the view that, given the appropriate environment infant immune cells can behave similarly to adult (11, 13–15, 58). While it is generally believed that the deficient response of very young children to influenza infection is due to antigen inexperience in T or B cells, the difference in the immune response could also be determined by cells other than immune cells that contribute to shape the adaptive immune response mediated by T or B cells.

For instance, epithelial cells (as well as endothelial cells) can produce cytokines and other factors that can modulate the type or strength of CD4 and CD8 cell mediated immune responses. Lung epithelial cells are the main cell target of influenza virus, as they express high levels of sialic acid on the cell surface to which influenza binds, subsequently enters, and replicates (19). However, lung epithelial cells can also orchestrate the innate anti-viral immune response. Influenza virus replication induces expression and production of type I IFN that acts as an anti-viral factor (39). Furthermore, lung epithelial cells are known to be able to produce different inflammatory cytokines (e.g. IL-33, IL-1, IL-6) that can then have an effect on the adaptive immune response (19). Under physiologic conditions (not during exposure to an insult) lung epithelial cells from adult mice express high levels of IL-6 mRNA in contrast to the relative absence during of IL-6 expression in lung resident macrophages (38). IL-6 production and secretion by mouse and human lung epithelial cells is triggered during infection with influenza virus and other viruses such coronavirus (39, 59, 60). IL-6 plays a pivotal role in dictating the types of CD4 and CD8 cell responses. Further, IL-6 has been shown to promote differentiation of CD4 cells into Th2 and Tfh cells that can then, by secreting IL-21 and IL-4, enhance isotype switching and antibody production in B cells (40, 61, 62). In influenza virus infection, IL-6 triggers IL-21 production by CD4 cells and IL-21 is essential for virus antibody response (40). In addition, we have also shown that IL-6 makes CD8 cells to become helpers of B cells through induction of IL-21. During influenza virus, CD8 cells in the lung but not in lymph nodes produce IL-21 and this effect requires IL-6 (55). Thus, the difference in IL-6 production by lung epithelial cells between infant and adults could account for the difference in T and B cell responses to influenza virus in the lung.

In this study, our gene profile analyses revealed marked differences the expression of immune regulators in lung epithelial cells from infants as compared to adult mice. Our pathway analysis has revealed IL-6 as a highly selective gene that is lower in pre-weanling epithelial cells. Interestingly, expression of other cytokines that play a role in antiviral immune responses (e.g. type I IFN), regulation of the adaptive immune response (e.g. IL-33) or the innate immune response (e.g. IL-18) was not different between infants and adult mice, further highlighting the potential significance of the differential expression of IL-6 that we observed. The evidence pointing to IL-6 expression in lung epithelial cells as being developmentally regulated could have a major impact on the understanding of childhood immune responses to pathogenic influenza viruses, but also to other respiratory viruses e.g., those producing SARS.

Since our gene expression profiling reveals a marked reduction (more than 3-fold lower) in the IL-6 expression in lung epithelial cells from infants relative to adult mice, it is possible that the lower IL-6 levels in the lungs could be responsible for the increased susceptibility. In this regard, adult mice lacking IL-6 or IL-6R die in response to sublethal dose of influenza virus in part due to the reduced number of neutrophils in the lung to mediate virus clearance (41). In addition, IL-6 can ameliorate acute lung injury in influenza virus infection in mice by promoting tissue repair (63). In humans, the systemic treatment with tocilizumab, the blocking anti-IL-6R antibody approved for treatment of rheumatoid arthritis, has also been shown to increase the risk to respiratory virus infection (64). Therefore, although exaggerated levels of IL-6 have been observed in adult patients with acute respiratory distress syndrome due to massive lung tissue damage (65, 66), IL-6 also likely provides protection from infections with influenza virus and other respiratory viruses (66).

Current influenza vaccination formulations include a live attenuated virus given intranasally, and an inactivated virus administered *via* intramuscular injection (26). Each has variable protection, depending on the formulation, the year of production, and the population vaccinated (27). In addition, the live-attenuated vaccine is not approved for very young children less than 2 years of age. Thus, this population remains at high risk every season. Here we show that a vaccine with inactive influenza virus provides protection to ~15 day-old mice when administered intranasally, suggesting a potential alternative option of vaccination for this highly susceptible population of infant children. Interestingly, our data also show that the protective effect of the intranasal immunization with the inactive influenza virus in infant mice is dependent on IL-6 since it fails to provide protection in IL-6 deficient mice. In contrast to i.n. administration, intramuscular administration of the inactive influenza virus did not provide protection in infant mice, stressing the importance of the intranasal route over the intramuscular route at this dose. The induction of IL-6 by the i.n. vaccine with the inactive virus suggested that this IL-6 may come from lung epithelial cells, although future studies will be needed to further demonstrate that this is the case. Using a commercially formulated multivalent vaccine for the 2012/2013 season, it has been reported that i.m. vaccination produced a protective response in infant mice (67). It is possible that the mix of influenza virus strains (H3N2 in addition to H1N1) in that particular seasonal vaccine could trigger a stronger immune response. However, correlating with our studies in infants, i.m. administration of inactive influenza virus in adult mice also induces a limited antibody response (40). The superior efficacy of the inactive virus vaccine when administered i.n. further reinforces the relevance of the environment where the immune response takes place. Most approved antiviral vaccines are based on their ability to induce antibody production. There is evidence that LAIV may trigger some T cell mediated protection (68, 69) although it has not been fully demonstrated. Intriguingly, here we show a reduction in the frequency of activated CD4 and CD8 cells in the lung in immunized IL-6 deficient mice relative to WT mice. Thus, it is possible that the lower efficacy of our inactive influenza viral vaccine in the IL-6 deficient mice could be due to impaired memory CD4/CD8 T cell response and the subsequent antibody response.

This investigation posits that vaccination of the infant can generate a protective response against lethal challenge if the correct environment is achieved. Our studies are in keeping with previous studies suggesting that immunization of infants against differing antigens, depending on the right source and format can lead to T cell activation in the relevant tissue and/or systemic antibody production (70). The critical role played by IL-6 in these studies when taken in context are consistent with the idea that environment matters in the development of infant immunity. Further understanding and the ability to harness specific tissue environments may aid in developing strategies for protection of the very young.

## Data Availability Statement

The raw data supporting the conclusions of this article will be made available by the authors, without undue reservation.

## Ethics Statement

The animal study was reviewed and approved by University of Vermont Institutional Animal Care and Use Committee.

## Author Contributions

EB designed and helped perform experiments, managed, analyzed, and interpreted the data, and wrote/edited the figures and manuscript. JK helped do gene expression analysis studies. BT helped perform experiments, as did KP and KK. LH provided PR8 and helped with data analysis and editing. MR helped perform experiments, analyzed, and interpreted the data, and wrote and edited the manuscript. All authors contributed to the article and approved the submitted version.

## Funding

Support for this work came from NIH P30GM118228 to the Vermont Center for Immunology and Infectious Disease, P30GM103532 to the Vermont Lung Center, the University of Vermont College of Medicine New Research Initiative Program, the UVM Flow Cytometry and Cell Sorting Facility, and the Department of Obstetrics, Gynecology and Reproductive Sciences. MR was also supported by NIH R56 AI116255 and R01 AI051454. LH and BT are supported by the University of Connecticut Center on Aging. JK is supported by the University of Colorado Cancer Center Bioinformatics/Biostatistics Shared Resources, supported by NIH P30CA046934. Research reported herein was also supported by an Institutional Development Award (IDeA) from the National Institute of General Medical Sciences of the National Institutes of Health under grant number P20GM103449, The Vermont Genetics Network.

## Conflict of Interest

The authors declare that the research was conducted in the absence of any commercial or financial relationships that could be construed as a potential conflict of interest.
