# Supplementary material for: Protective Intranasal Immunization Against Influenza Virus in Infant Mice Is Dependent on IL-6
Source: Front Immunol. 2020 Oct 28;11:568978. doi: 10.3389/fimmu.2020.568978 (PMC7656064; doi:10.3389/fimmu.2020.568978)
Supplement: Supplementary file 1 [file DataSheet_1.zip › Supplemental Figure 1.pdf]

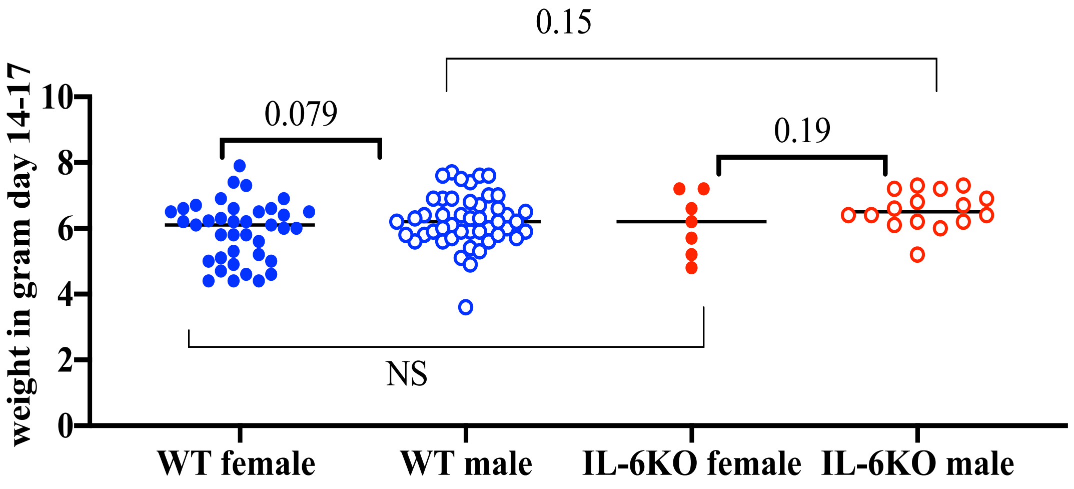

Figure S1. Comparison of pup weights at 2 weeks. Individual pup weights (grams) for WT and IL6KO mice at 14-16 days of life were recorded and analyzed by ANOVA with multiple comparisons. Each symbol represents a pup. Data from eight cohorts is shown.
