# Supplementary material for: Protective Intranasal Immunization Against Influenza Virus in Infant Mice Is Dependent on IL-6
Source: Front Immunol. 2020 Oct 28;11:568978. doi: 10.3389/fimmu.2020.568978 (PMC7656064; doi:10.3389/fimmu.2020.568978)
Supplement: Supplementary file 1 [file DataSheet_1.zip › Supplemental Figure 2.pdf]

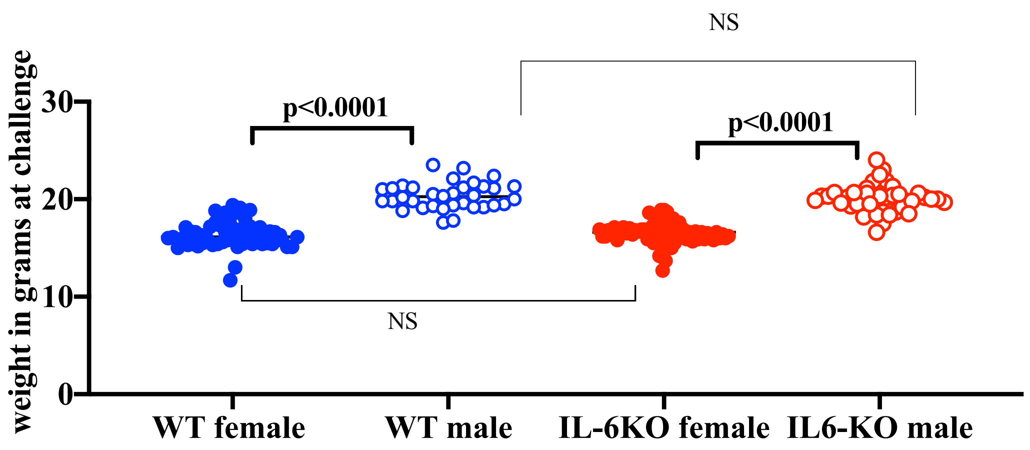


**Figure S2. Weights of mice undergoing lethal challenge.** Individual pup weights (grams) for WT and IL6KO mice at challenge in 15 total cohorts was recorded and analyzed by ANOVA as in S1 above. Each symbol represents a pup.
