# Supplementary material for: Protective Intranasal Immunization Against Influenza Virus in Infant Mice Is Dependent on IL-6
Source: Front Immunol. 2020 Oct 28;11:568978. doi: 10.3389/fimmu.2020.568978 (PMC7656064; doi:10.3389/fimmu.2020.568978)
Supplement: Supplementary file 1 [file DataSheet_1.zip › Supplemental Figure 3.pdf]

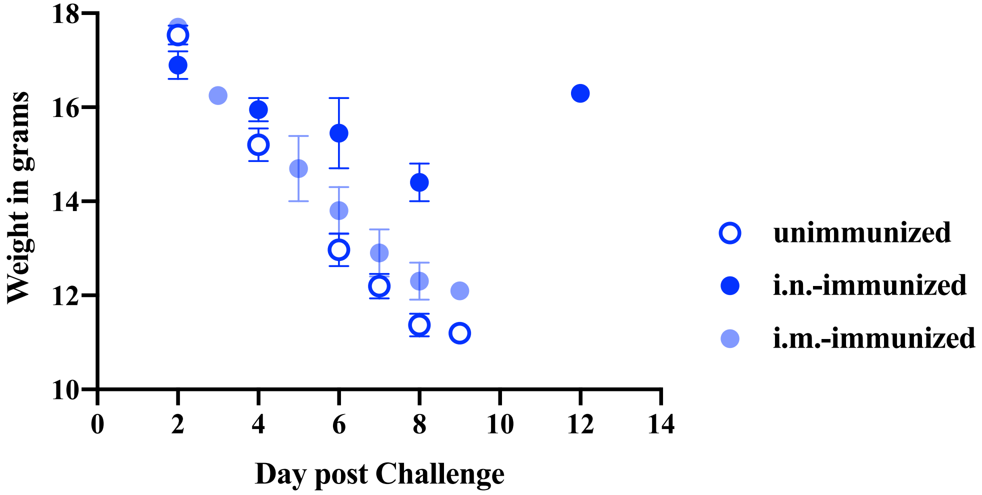


**Figure S3. Representative examples of WT females undergoing immunization and challenge.** WT female pups were immunized and subsequently challenged as in Figure 2. Y axis, weight at challenge. X axis, time post challenge. Shown is representative data from 3 cohorts to demonstrate the course of weight loss and recovery. Dark blue, mice given intranasal immunization. Open symbols, u.n.-immunized mice. Lt Blue symbols, i.m. immunized mice. Mean +/- Standard error of 3 mice per group is shown.
