# Supplementary material for: Protective Intranasal Immunization Against Influenza Virus in Infant Mice Is Dependent on IL-6
Source: Front Immunol. 2020 Oct 28;11:568978. doi: 10.3389/fimmu.2020.568978 (PMC7656064; doi:10.3389/fimmu.2020.568978)
Supplement: Supplementary file 1 [file DataSheet_1.zip › Supplemental Figure 4.pdf]

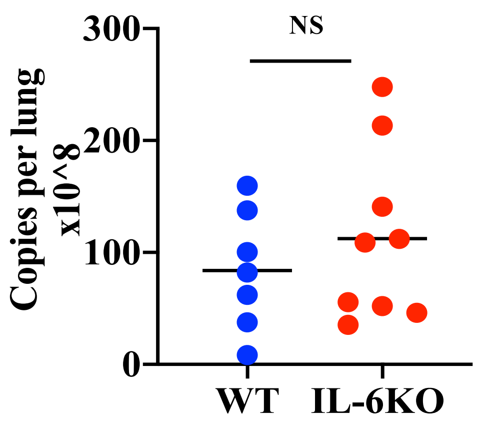

Figure S4. Lung viral load in nasally immunized and lethally challenge WT and IL6KO mice. Mice were immunized intranasally and challenged with $6x10^3$ EIU three weeks later. Six days after challenge, mice were euthanized and whole lung was used to determine viral load by PCR as noted above. Hypothesis testing utilize the t test. NS, not significant.
