# Supplementary material for: Protective Intranasal Immunization Against Influenza Virus in Infant Mice Is Dependent on IL-6
Source: Front Immunol. 2020 Oct 28;11:568978. doi: 10.3389/fimmu.2020.568978 (PMC7656064; doi:10.3389/fimmu.2020.568978)
Supplement: Supplementary file 1 [file DataSheet_1.zip › Supplemental Figure 5.pdf]

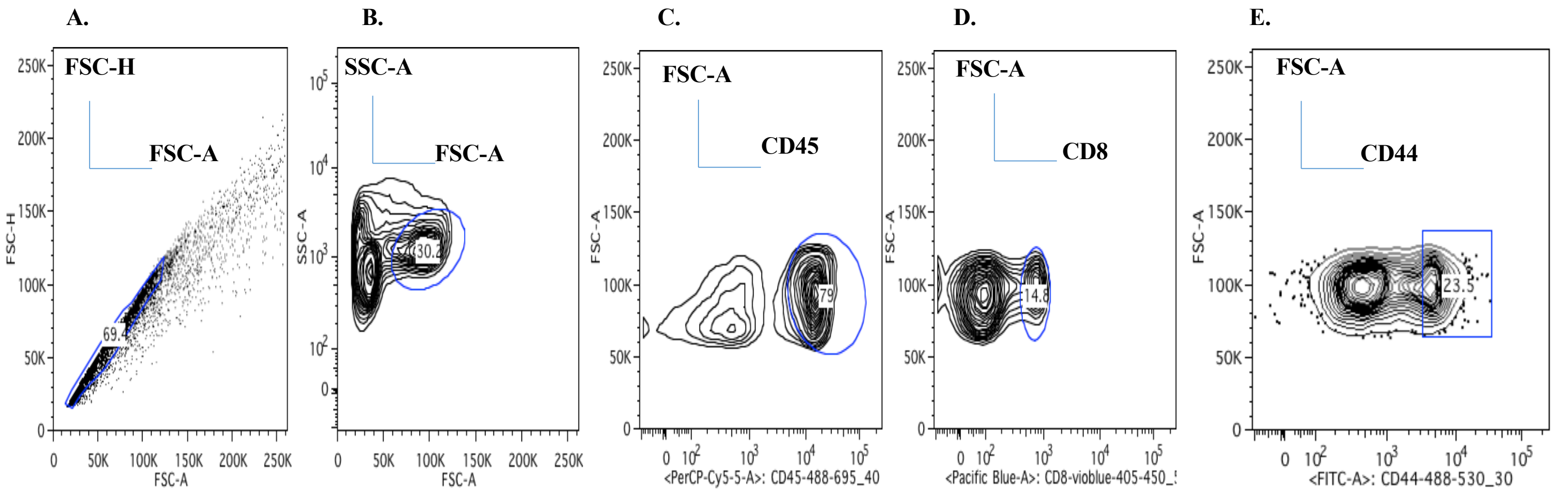

Figure S5. Flow cytometry schema for analysis of lung tissue. Single cell suspensions of homogenized whole lung were prepared, cleared of erythrocytes and stained with antibodies for CD45, CD4, CD8, and CD44 and then submitted for flow cytometry. Analysis included gating for singlets (**A**), Live leukocytes (**B**), CD45 (**C**), CD4 or 8 **(D)** and CD44^{hi} **(E).**
