# Supplementary material for: Protective Intranasal Immunization Against Influenza Virus in Infant Mice Is Dependent on IL-6
Source: Front Immunol. 2020 Oct 28;11:568978. doi: 10.3389/fimmu.2020.568978 (PMC7656064; doi:10.3389/fimmu.2020.568978)
Supplement: Supplementary file 1 [file DataSheet_1.zip › Supplemental Table 1.pdf]

~age(day)	dose(EIU)	% died	# survived	# died	*dose/weight
10	60	0	6	0	11
10	140	57.1	3	4	26
10	160	57.1	3	4	30
10	200	85.7	1	6	40
15	200	65	7	13	34
15	200	75	1	3	34
25	300	0	3	0	28
25	900	25	3	1	85
25	1800	100	0	4	170
35	3000	33.3	2	1	198
35	3000	0	3	0	186
35	3000	0	3	0	173
34	6000	50	1	1	382
38	6000	78	2	7	368
42	6000	50	2	2	353

Supplemental Table 1. Summary of unimmunized WT Females. Summary of overall result of intranasal infection with live PR8 in unimmunized female mice by age (days of life) and dose (EIU) in ~20 cohorts. Red boxes indicate doses used for 10,15,25, and 35-day-old mice used in Fig. 1. Asterisk: dose/average weight in grams.
