# Supplementary figures and images for: Protective Intranasal Immunization Against Influenza Virus in Infant Mice Is Dependent on IL-6
Source: Front Immunol. 2020 Oct 28;11:568978. doi: 10.3389/fimmu.2020.568978 (PMC7656064; doi:10.3389/fimmu.2020.568978)

**Figures S6 (A-E).** Larger versions of the heat map data in **Figure 3**.

Cytosolic DNA sensing  
p= 0.0004

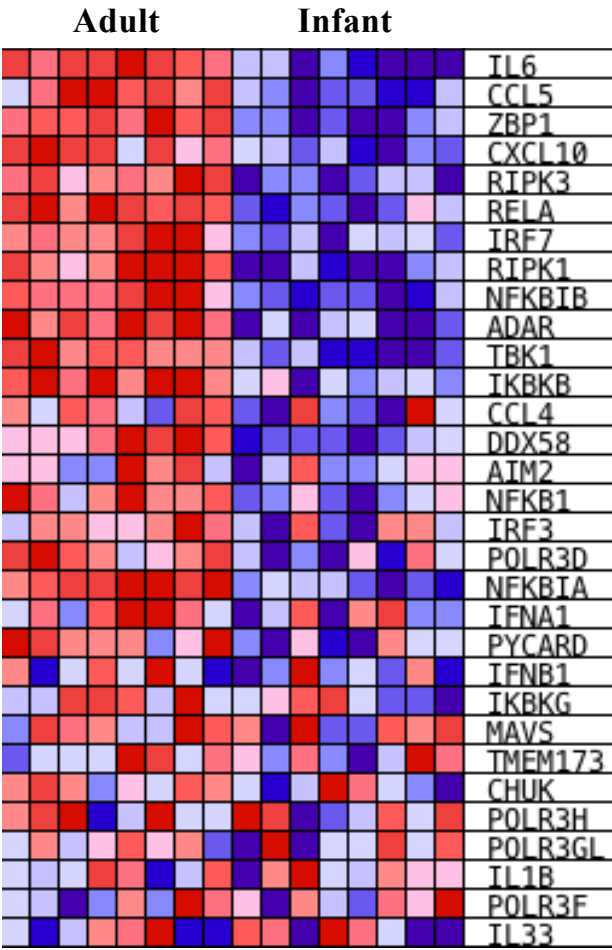

**Figure S6A**

Supplement: Supplementary file 1 [file DataSheet_1.zip › Supplemental Figure 6A.pdf]

Jak/Stat pathway  
p= 0.0011

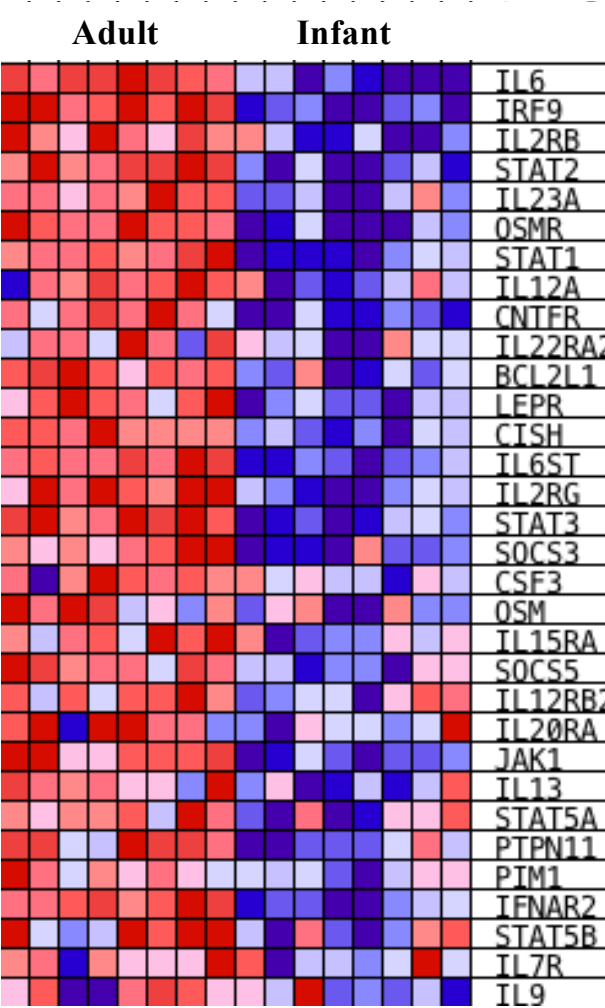

Figure S6B

Supplement: Supplementary file 1 [file DataSheet_1.zip › Supplemental Figure 6B.pdf]

Cytokine/ Cytokine Receptor  
p= 0.015

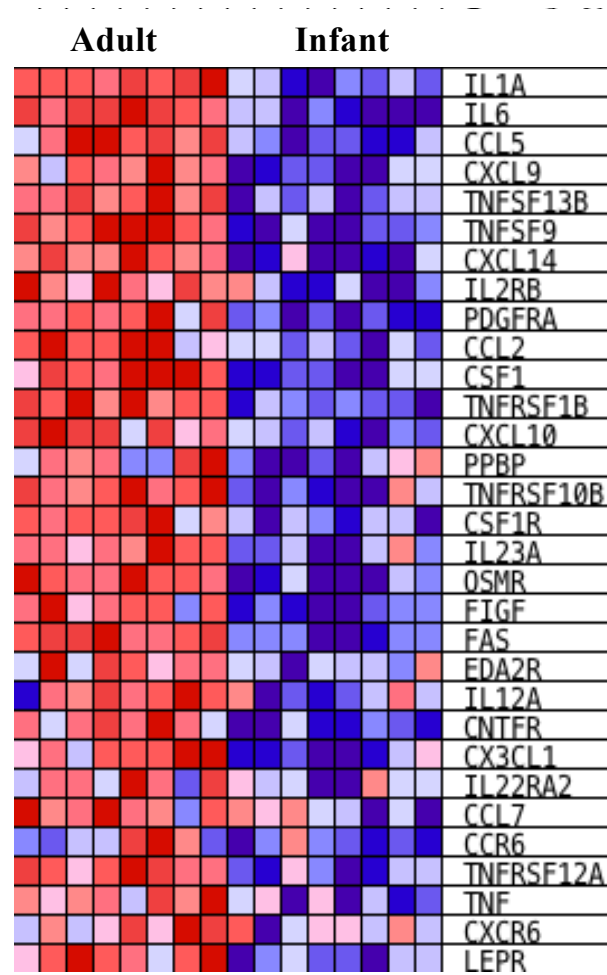

Figure S6C

Supplement: Supplementary file 1 [file DataSheet_1.zip › Supplemental Figure 6C.pdf]

TLR pathway  
p= 0.0007

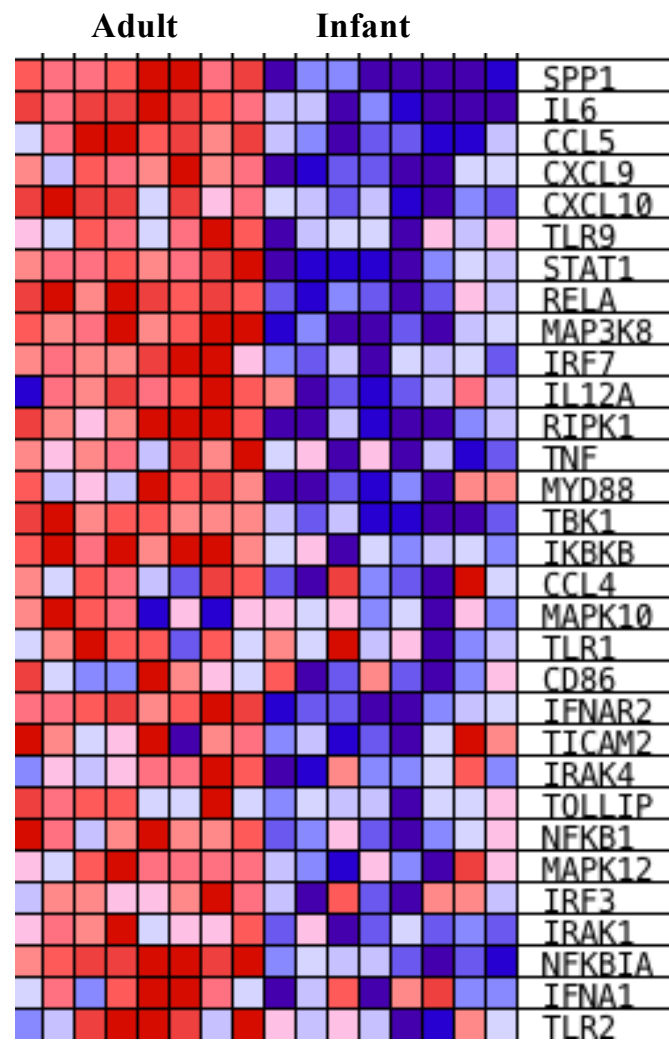

Figure S6D

Supplement: Supplementary file 1 [file DataSheet_1.zip › Supplemental Figure 6D.pdf]
